# Preparing *Arabidopsis thaliana* root protoplasts for cryo electron tomography

**DOI:** 10.3389/fpls.2023.1261180

**Published:** 2023-09-22

**Authors:** Ingrid Berenice Sanchez Carrillo, Patrick C. Hoffmann, Teura Barff, Martin Beck, Hugo Germain

**Affiliations:** ^1^ Department of Chemistry, Biochemistry, and Physics, Université du Québec à Trois-Rivières, Trois-Rivières, QC, Canada; ^2^ Department of Molecular Sociology, Max-Planck-Institute for Biophysics, Frankfurt, Germany; ^3^ Institute of Biochemistry, Goethe University Frankfurt, Frankfurt, Germany

**Keywords:** structural biology, Arabidopsis thaliana, protoplasts, cryo-EM, cryo-ET

## Abstract

The use of protoplasts in plant biology has become a convenient tool for the application of transient gene expression. This model system has allowed the study of plant responses to biotic and abiotic stresses, protein location and trafficking, cell wall dynamics, and single-cell transcriptomics, among others. Although well-established protocols for isolating protoplasts from different plant tissues are available, they have never been used for studying plant cells using cryo electron microscopy (cryo-EM) and cryo electron tomography (cryo-ET). Here we describe a workflow to prepare root protoplasts from *Arabidopsis thaliana* plants for cryo-ET. The process includes protoplast isolation and vitrification on EM grids, and cryo-focused ion beam milling (cryo-FIB), with the aim of tilt series acquisition. The whole workflow, from growing the plants to the acquisition of the tilt series, may take a few months. Our protocol provides a novel application to use plant protoplasts as a tool for cryo-ET.

## Introduction

Understanding organelles’ spatial distribution and macromolecules’ cellular structure is fundamental to cell biology. Over the years, several techniques aiming to improve this understanding have been successfully applied to yeast and various mammalian cells, notably using electron cryo microscopy (cryo-EM) and cryo electron tomography (cryo-ET) approaches, which have provided unprecedented resolution (Oikonomou and Jensen, 2017; Pfeffer and Mahamid, 2018; Kühlbrandt, 2022; Young and Villa, 2023). Despite its many advances, applying EM techniques has been a challenge for plant biologists due to the thickness of plant tissues, cell and organelle size, presence of a cell wall, and preservation and subsequent handling of the sample for analysis (Otegui and Pennington, 2018; Liu et al., 2020). The visualization and localization of cellular components, organelle morphology, and analysis of individual structures have been possible thanks to the progress that light and electron microscopy approaches have undergone over the years (Antony et al., 2013; Benjin and Ling, 2020; Cooper, 2000). On the one hand, fluorescence microscopy, with the use of a larger selection of better fluorophores, has made it possible to follow cellular processes and locate molecules of interest while imaging living cells. With its much higher spatial resolution, EM has allowed for studying different organisms at a much higher resolution, while obtaining information on cellular components that, combined with data from other methods, provides a valuable understanding of essential cellular processes (Cohen et al., 2002; O'Toole et al., 2002; Geimer and Melkonian, 2005; Herold et al., 2009; Kurth et al., 2010; Weiner et al., 2021; Kaplan et al., 2022).

Amongst the various EM approaches that can be applied to investigate biological specimens, cryo-EM has allowed scientists to visualize samples that are frozen-hydrated in their near-native (Murata and Wolf, 2018; Zhang, 2019). Therefore, through rapid freezing, it is possible to obtain structural preservation while maintaining the integrity of cells, organelles, and molecular assemblies (Beck and Baumeister, 2016). To study these frozen-hydrated samples, one of the structural determination methods that can be used is cryo-ET combined with subtomogram averaging (STA) (Wan and Briggs, 2016). Furthermore, it is necessary to consider that sample thickness represents a limiting factor for cryo-ET, effectively restricting this technique to samples thinner than 0.3-0.5 µm (Mahamid et al., 2015; Beck and Baumeister, 2016). To overcome this issue, vitrified specimens can be thinned using a focused ion beam microscope under cryogenic conditions (cryo-FIB) (Rigort and Plitzko, 2015). These methods together allow for achieving reconstructions that can reach sub-nanometer resolution (Wang et al., 2021; Zimmerli et al., 2021; Hoffmann et al., 2022; Lucas et al., 2022; Mosalaganti et al., 2022; Gemmer et al., 2023; Khavnekar et al., 2023).

While it has been possible to apply classical EM and ET techniques to study cellular plant processes (Reyes et al., 2011; Nicolas et al., 2017; Liang et al., 2018; Wang et al., 2019), few publications have shown that cryo-FIB milling and cryo-ET can be performed in plants and algae. The best-studied organism using these methods is *Chlamydomonas reinhardtii*, a small unicellular green alga, for which chloroplast architecture (Engel et al., 2015a), Golgi intracisternal protein arrays (Engel et al., 2015b), the native structure of COPI coats (Bykov et al., 2017), 26S proteasome complexes tethering to nuclear pore complexes (NPCs) (Albert et al., 2017), *in situ* structural studies of membrane proteins (Schaffer et al., 2017), *in situ* architecture of NPCs (Mosalaganti et al., 2018), centriole architecture (Klena et al., 2020), and thylakoid’s individual protein complexes (Wietrzynski et al., 2020) have been revealed. More recently, cryo-FIB milling has been successfully applied using white onion (*Allium cepa*) epidermal cell wall peels to reveal the organization of cellulose fibers *in situ* (Nicolas et al., 2022 *).* Likewise, cryo-lift-out in combination with cryo-ET have been employed effectively for studying tip-vesicles (TVs) in pollen tubes to better understand the function of TVs in pollen tubes (Liu et al., 2020). However, to date, the *in situ* use of cryo-FIB together with cryo-ET in vascular plant cells for revealing the structure of macromolecular assemblies and organelles in their near-native state in higher plants has not been achieved.

Here we describe a protocol for preparing plant protoplasts for cryo-FIB milling that enables the acquisition of tilt series. Plant protoplasts are isolated cells without cell walls (Yoo et al., 2007) that have been widely used as a versatile cell-based system to investigate plant cell reprograming (Pasternak et al., 2020), transient and early events using transient expression systems (Yoo et al., 2007), screening of proteins involved to a specific abiotic (Chen et al., 2010; Wehner et al., 2011) or biotic stress (Asai et al., 2002; He et al., 2006; Boudsocq et al., 2010), auxin signaling and responses (Worley et al., 2000; Böttner et al., 2009), protein-protein interaction (Halter et al., 2014; Yeh et al., 2015; Gong et al., 2019; Ye et al., 2019), protein trafficking and localization (Asai et al., 2002; Ehlert et al., 2006; Underwood et al., 2017; Menzel et al., 2019), protoplast regeneration and plant breeding (Reed and Bargmann, 2021), and single-cell analysis (Efroni et al., 2015; Ryu et al., 2019; Xu et al., 2021; Zong et al., 2022), among others. Even if the protoplast isolation process involves eliminating the cell wall through an enzymatic process, it has been demonstrated that plant protoplasts retain their physiological responses, and original cellular and biochemical activities (Sheen, 2001; Yoo et al., 2007). While different protocols have been established for using protoplasts for various purposes, we describe the adaptations needed for *in situ* cryo-ET studies of plant macromolecules in their near-native. Our protocol details the steps necessary to obtain protoplasts from root tissue, to vitrify them on cryo-EM grids, and to target intact protoplasts for cryo-FIB lamellae preparation for cryo-ET. The protoplasts can in principle also be obtained from other tissues by adapting the protoplast isolation specifications. Lamellae preparation is performed by focused gallium ion beam milling, comparable to established protocols for other cell types (Schaffer et al., 2015; Wagner et al., 2020). The use of protoplasts, which are of a size consistent with the sample thickness required for cryo-FIB milling, and the fact that they can be properly vitrified makes this protocol suitable for tilt series acquisition and it could subsequently be used for sub-tomogram averaging.

## Overview of the procedure

**Figure 1:**
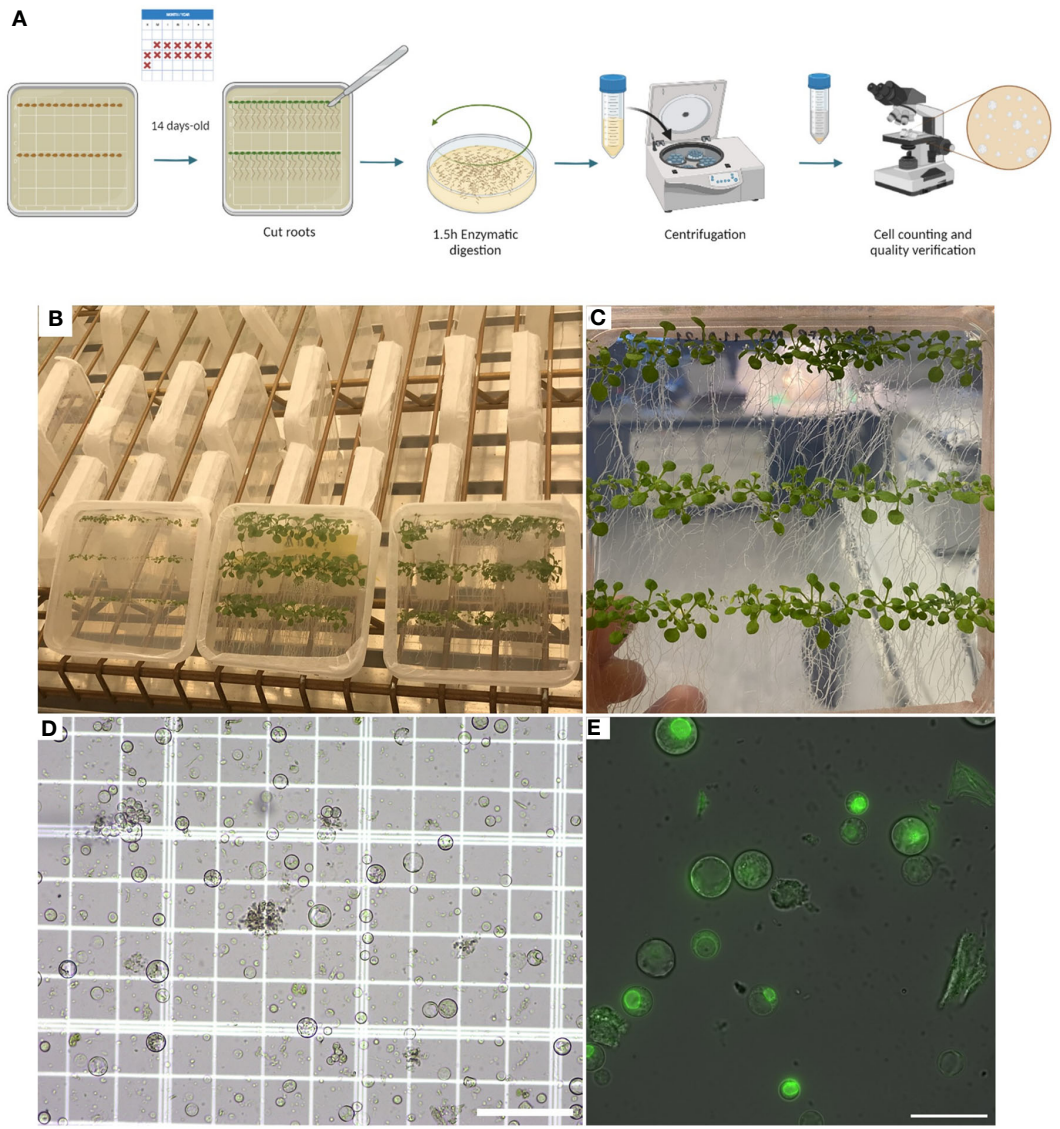
Plant growth and protoplast isolation (Steps 2–18). **(A)**, Diagram describing the workflow from growing Arabidopsis plants in petri dishes containing MS media with agar for isolating and verifying root protoplasts under a microscope. **(B)**, Photo showing how plants can be grown vertically for the roots to grow over the surface of the MS media with agar. **(C)**, Photo of 14-day-old *Arabidopsis* plants expressing RAE1-GFP grown vertically. **(D)**, Image of root protoplasts after digestion under a light microscope. **(E)**, Confocal fluorescence image of root protoplasts expressing RAE1-GFP. Scale bars: 50 µm **(D)** and 25 µm **(E)**.

Before isolating protoplasts from Arabidopsis’ roots, previously sterilized seeds should be grown in Murashige and Skoog Basal Medium (MS medium) with agar and Petri dishes must be placed vertically in the growth chamber. In this way, the plants will grow in a sterile manner, while the vertical position will cause the roots to grow at the surface of the growth media, allowing the user to have easy access to the roots once the plants are ready for use (Steps 1 and 2). After two weeks of growth, the roots can be easily cut and separated from the aerial part with a scalpel. Then, roots are immersed in the enzyme solution and cut into small pieces to start the digestion process of the plant cell wall (Steps 3-5). Once the digestion has been done, the isolated protoplasts must be filtered, spun, and washed for quantification and verification (**Figure 1**) (Steps 6-18) before proceeding to plunge freezing.

**Figure 2:**
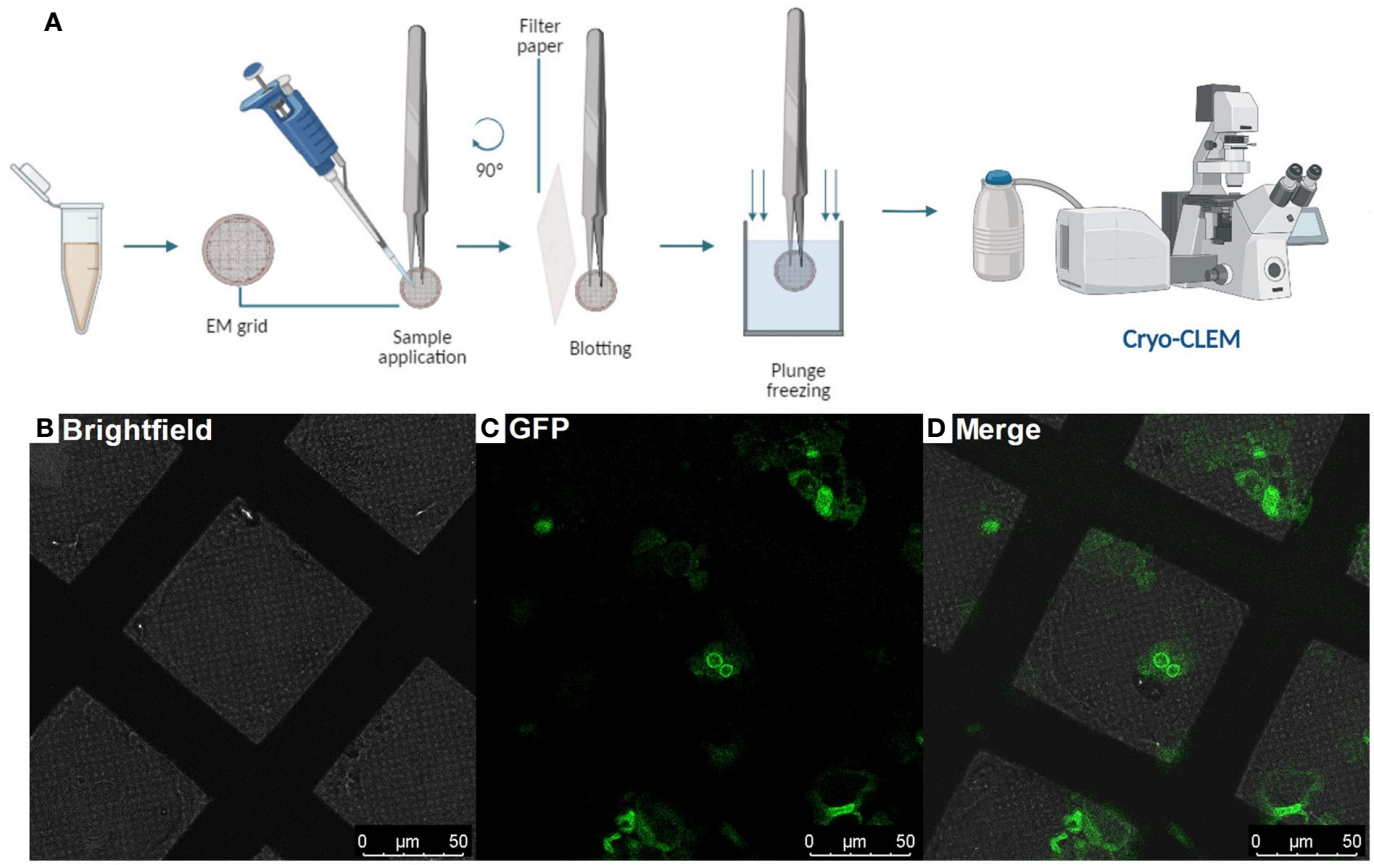
Protoplasts vitrification by plunge-freezing and Correlative Light and Electron Microscopy (Steps 19 - 29 and 40 - 47) **(A)**, Diagram showing the steps to follow after protoplast isolation: Plunge freezing procedure and observing the EM grids containing the root protoplasts under a cryo-CLEM, **(B-D)**, Cryo-Fluorescence Microscopy of a grid containing root-protoplasts from *Arabidopsis thaliana’s* RAE1-GFP transgenic line. Brightfield: field of bright light; GFP: Green fluorescence signal (green); Merge shows the overlay of the brightfield and GFP signal.

Plunge freezing (Steps 19-29) can be performed in liquid ethane with a plunger while adjusting the parameters according to the equipment’s model and specifications. Next, the EM grids must be clipped to continue with the workflow (Steps 30-39). Correlative Light and Electron Microscopy (CLEM) should be used to help identify intact protoplasts on the grids, and regions of interest that are labeled with a fluorophore using a light microscope fitted with a cryo-stage (Step 40-47) (**Figure 2**).

**Figure 3:**
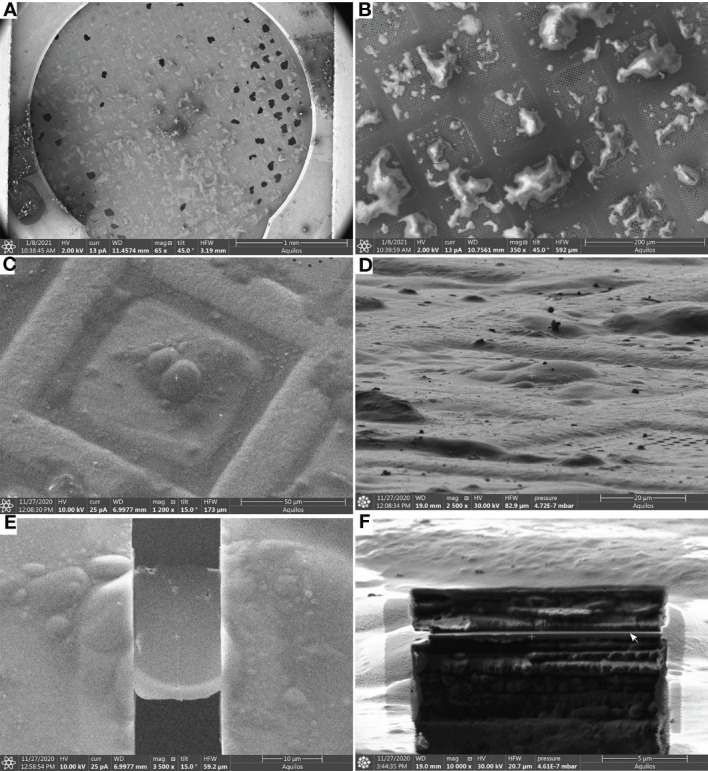
Lamella milling (Steps 48-56) **(A)**, Scanning Electron Microscope (SEM) view of an EM grid containing vitrified root protoplasts from *Arabidopsis thaliana* RAE1-GFP transgenic line. **(B, C)**, SEM images (top view) of root protoplasts before milling. **(D)**, FIB view of root protoplasts. **(E)**, SEM view of a cryo-FIB milled lamella, and **(F)**, FIB view of a lamella. The lamella front in **(F)** is indicated by a white arrow. Scale bars: 1 mm (a), 200 µm (b), 50µm (c), 20 µm (d), 10 µm **(E)**, and 5 µm **(F)**.

Once the target is identified, lamellae can be milled (Steps 48-56) from protoplasts on the EM grids in a dual-beam FIB/SEM microscope (**Figure 3**).

**Figure 4:**
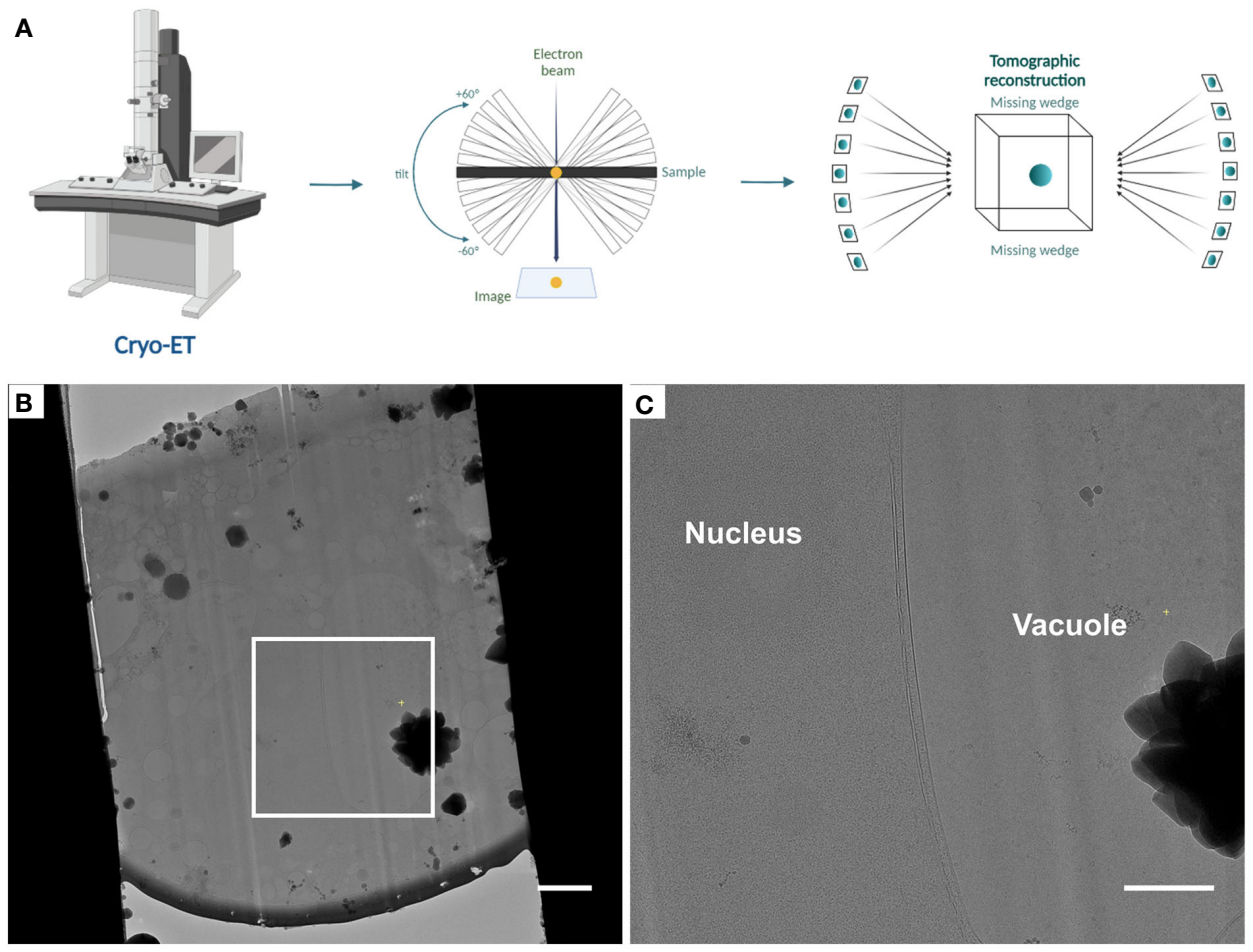
Automated tomogram acquisition and tomogram reconstruction (Steps 57 - 64) **(A)**, Diagram representing the steps of tomogram acquisition and tomogram reconstruction. **(B)**, Cryo-TEM overview of lamellae after fine milling to a thickness of ~ 180 - 200 nm, the white box indicates the area shown in **(C)**. **(C)**, Higher magnification cryo-TEM overview. Scale bars: 2 µm **(B)** and 1 µm **(C)**.

Finally, cryo-ET tilt series are acquired from these lamellae following established procedures (Steps 57-60) (**Figure 4**). Tomogram reconstruction was done using IMOD (Steps 61-64) and should be adjusted based on the needs and objectives of the respective study.

## Level of expertise

The protocol presented here requires prior knowledge about growth conditions of *Arabidopsis thaliana*, as well as protoplast handling and EM sample preparation at cryogenic temperatures. Thus, we presume that the researchers who would like to apply this workflow are familiar with light microscopy as well as cryo-ET sample preparation. Likewise, researchers should already be acquainted with the equipment and tools necessary to carry out the steps described here.

## Applications of the method

This protocol describes a step-by-step procedure that allows the user to isolate plant protoplasts and prepare them for cryo-EM and cryo-ET. The application of *in situ* cryo-ET and its supplementary approaches to vascular plants was initially considered challenging due to their complexity and size (Otegui and Pennington, 2018; Liu et al., 2020). Our workflow introduces a possibility to carry out cryo-FIB sample preparation for cryo-ET studies on higher plants by using fresh plant protoplasts that can be obtained from different plant tissues and species.

This protocol can be applied to reveal the native structure of macromolecular complexes, proteins, and organelles in plant cells. To achieve this goal, modifications to this protocol could be made to expand its application.

Our protocol is strongly focused on the sample preparation of protoplasts for cryo-ET, we do not aim to describe reconstruction in detail. These steps can be performed with various software and protocols may vary depending on the scientific question asked. Nevertheless, we included these steps to give a complete picture from growing plants to analyzing data.

## Comparison with other methods

This protocol was developed to apply cryo-ET approaches using plant protoplasts for the visualization of organelles and macro-molecular complexes in their native environment. The application of the approaches mentioned in this protocol provides a new area of opportunity for *in situ* studies of complexes and organelles in plant cells. It is important to control the concentration of protoplasts to be plunge-frozen on a single EM grid. By taking care of this aspect, a density of protoplasts that can be analyzed is ensured, avoiding crystaline ice formation in thick samples that would harm molecular preservation. Other alternative methods have been used previously for the study and modeling of plant organelles. These alternatives include the application of cryo-immobilization under high pressure, followed by freeze-substitution (Austin et al., 2005; Otegui et al., 2006; Wilson and Bacic, 2012; Takeuchi et al., 2016; Cui et al., 2019), and more recently, the waffle method (Kelley et al., 2022) which enables high-pressure freezing of thicker samples on grids for cryo-FIB milling. Although these approaches that do preserve native structures have provided valuable information about cellular structures in plants, some still use chemical fixatives and electron-dense stains to improve contrast, which are not compatible with native preservation needed for *in situ* structural biology. On the other hand, waffle and lift-out approches usually require more experienced user interaction and more microscope time during sample preparation for their application.

In comparison, our protocol allows for the observation of frozen-hydrated samples close to their near native state without the use of chemicals or staining with heavy metals. Furthermore, it also offers the advantage of assessing protein structure inside plant protoplasts.

## Advantages and limitations

The advantages of this protocol comprise the ability to prepare protoplasts from higher plants for cryo-EM and cryo-ET, while preserving the cellular structure. Among the advantages of our protocol, is the possibility to isolate protoplasts without requiring aseptic operations. This protocol can be easily implemented in almost any laboratory with access to a state-of-the-art EM facility. Similarly, it is possible to adapt the enzymatic solution for cell wall digestion, digestion time, sample to be used, and plunge freezing conditions and thus to apply this protocol to various plant sources. Another relevant point to consider is that in this work we used roots of *Arabidopsis thaliana* plants to obtain protoplasts. Therefore, it is essential to consider that we expected to have a heterogeneous population of cells. The heterogeneity of cell population could vary according to the type of organ used.

This workflow may present some disadvantages that should be considered before application. For example, while it is possible to find several efficient established protocols to successfully isolate protoplasts from model plants and crops, it is not feasible to isolate enough viable protoplasts from all organs and tissues of all plants due to species specificity constraints (Ren et al., 2021). However, the number of viable protoplasts on the grid directly affects EM grid quality as well as sample throughput and deviations of these parameters may make it difficult to obtain lamellae. Our protocol presents a powerful workflow for *in situ* structural studies of vascular plant cells, yet protoplasts are isolated cells outside of the context of tissue environment, which needs to be considered with respect to cell physiology and impact for the underlying cellular architecture.

**MATERIALS:**

REAGENTS

- Murashige and Skoog Basal Medium (Sigma-Aldrich, cat. no. M5519)

- Agar (Bio Basic, cat.no. FB0010)

- KOH (Millipore Sigma, cat. No. 105033)

- 0.2 M 4-morpholineethanesulfonic acid (MES, pH 5.7; FisherBiotech, cat. no. BP300)

- 0.8 M mannitol (Acros, cat. no.125340010)

- 1 M CaCl_2_ (Bio Basic, cat. no. CT1330)

- 2 M KCl (Fisher scientific, cat. no. P330-500)

- 2 M MgCl_2_ (Fisher Scientific, cat. no.M-20944)

- 154 mM NaCl (Fisher Scientific, cat. no. S271)

- Tris Base (Millipore Sigma, cat. no. 648310)

- HCl (Millipore Sigma, cat. no. 258148)

- β-Mercaptoethanol (Fisher Scientific, cat. no. BP176-100)

- BSA (Roche, cat. no. 10 735 086 001)

- Cellulase from (*Trichoderma* sp., Sigma-Aldrich, cat. no. C0615-1G)

- Pectinase from (*Aspergillus niger*, Sigma-Aldrich, cat. no. P4716)

- Concanavalin A (Sigma Aldrich, cat. no. C2010)

- Enzyme solution (see REAGENT SETUP)

- W5 solution (see REAGENT SETUP)

BIOLOGICAL MATERIALS

- *Arabidopsis* accessions: Col-0, RAE1-GFP (Tamura et al., 2010)

EQUIPMENT

- Square Petri Dishes (Fisher Scientific, cat no. FB0875711A)

- Sorvall Legend XFR Centrifuge (Thermo Fisher Scientific, cat. no. 75004541)

- 0.2-μm Syringe filter – Sterile (Fisher Scientific, cat. no. 09-719C)

- Sterile Single Use Carbon Blades (Lance, cat. no. 1500321)

- Petri dish with clear lid (Fisher Scientific, cat. no. FB0875712)

- Micropore (3M, cat. no. 1530-1)

- Sterile Cell Strainer 70 µm Nylon Mesh (Fisher Scientific, cat. no. 22-363-548)

- Improved Neubauer 0.1-mm-deep hemacytometer (Hausser Scientific, cat. no. 0267110)

- OMAX 40-2500X LED Digital Trinocular Compound Microscope USB Camera (OMAX, cat. No. M83EZ-SCP-C03S)

- Fluorescence microscope (Nikon microscope Ti2 Eclipse or comparable)

- 35 mm microscopy dishes, no. 1.5 Coverslip, 14 mm Glass Diameter, uncoated (Mattek)

- 2/1 Holey Carbon-coated SiO_2_ 200-mesh Gold grids (Quantifoil Micro Tools GmbH, catalog number: Q2100CR1)

- Tweezers (Dumont 3, 5)

- Glass microscopy slides

- Glass Petri dishes

- Glow Discharge Cleaning System (PELCO easiGlow)

- Leica EM GP2 plunger (Leica Microsystems)

- Whatman filter paper grade 1

- Cryo grid boxes (custom-made) or (Thermo Fisher Scientific)

- Autogrids with FIB cutout (MPI Martinsried or Thermo Fisher Scientific)

- C-clips (Thermo Fisher Scientific)

- C-clip insertion tools (Thermo Fisher Scientific)

- EM Cryo CLEM system (Leica Microsystems)

- Aquilos Cryo-Focused Ion Beam - Scanning Electron Microscope (Thermo Fisher Scientific)

- Titan Krios G2 Cryo-Transmission Electron Microscope (Thermo Fisher Scientific), equipped with a BioQuantum-K3 (Gatan)

REAGENT SETUP

Enzyme solution:

Prepare a solution containing 0.4 M mannitol, 20 mM MES (pH 5.7), 20 mM KCl, 1.5% (wt/vol) cellulase, and 0.3% (wt/vol) macerozyme. Use 1 M Tris/HCl pH 7.5 to adjust the pH of the enzyme solution to 5.7. Warm the solution at 55°C for 10 minutes and cool to room temperature. Then, add 0.1% (wt/vol) of BSA, 10 mM of CaCl_2_, and 5 mM of β-mercaptoethanol. The resulting enzyme solution should have a clear, light brown color. Pass the final enzyme solution through a 0.2 μm syringe filter into a Petri dish. Generally, 5 mL of enzyme solution can be used per 350 seedlings.

**CRITICAL: The enzyme solution must be freshly made every time.**

W5 solution:

Prepare a solution containing 154 mM NaCl, 125 mM CaCl_2_, 5 mM KCl, and 2 mM MES (pH 5.7). The final W5 solution can be kept at room temperature.

**PROCEDURE**

Plant growth – Timing 2 weeks

1. Surface sterilize *Arabidopsis* seeds with 70% ethanol for 2 minutes, followed by 7% bleach and 0.1% Triton X-100 for 5 minutes. Then, wash the seeds 4 - 6 times with sterile water and stratify them in the dark for 48 hours at 4°C.

**CRITICAL STEP: Prolonged exposition to ethanol or bleach during this step may affect seed germination**.

2. In a biological safety cabinet, place seeds at a density of approximately 50 seeds per Petri box, in a straight line, over plant growth media consisting of ½ Murashige and Skoog Basal Medium supplemented with 1% agar and adjusted to pH 5.6-5.8 with KOH. Seal the Petri boxes using Micropore tape. Position the squared Petri boxes vertically under a long-day photoperiod (16h of light, 8h of dark) at 23 °C, with an average light intensity of 120 mmol/m-^2^/s^-1^ at the level of the rosette.

**CRITICAL STEP: the use of Micropore tape for sealing the Petri dishes keeps plates moist and sterile while allowing gas exchange.**

Protoplast Isolation – Timing 2-3 hours

Protoplasts from roots were isolated according to the procedure of Bargmann, B. and Birnbaum, K (Au - Bargmann and Au - Birnbaum, 2010)., with some modifications.

3. Harvest root tissue from Petri boxes containing 14-day-old plants with a scalpel and deposit them into the Petri dish containing 5 mL of enzyme solution.

4. Finely chop the roots until they are reduced into 1-2 mm pieces.

**CRITICAL STEP: It is essential to cut the roots when they are in the solution and not when exposed to air, as they may dry out and further affect the quality and quantity of protoplasts.**

5. Perform the enzymatic digestion from the chopped roots under agitation at 75 rpm for 1.5 h at room temperature.

**CRITICAL STEP: Digestion time needs to be empirically optimized and relies on the experimental aims and desired responses.**

6. After cell wall digestion, filter the solution through a 70 µm cell strainer nylon mesh into a new Falcon tube.

7. Add one volume of W5 solution, spin the protoplasts for 10 min at 500 x g, and then resuspend in cold W5 solution.

**CRITICAL STEP: It is important to be gentle with protoplasts; however, handling them with regular pipette tips is possible.**

8. For counting the protoplasts, place a glass cover slip over a Neubauer chamber (hemacytometer).

9. Using a pipette tip, gently mix the protoplast sample to ensure uniform cell dispersion.

10. Place the tip at the edge of the cover glass and use capillary action to the chambers of the Neubauer chamber.

11. Allow approximately one minute for the protoplasts to settle.

12. Examine each chamber at the proper magnification and count the number and quality of protoplasts in adequately sized grids.

13. The final protoplast concentration must be diluted to 500-650 protoplasts/µL in fresh W5 solution.

**CRITICAL STEP: The concentration of protoplasts per µL may vary according to the cell type. However, it is essential to note that a very low or very high concentration of protoplasts per µL may not be optimal for the following steps.**

**? TROUBLESHOOTING**

14. Use a wide-field fluorescence microscope to assess the fluorescence of the fluorophore-labeled target in the protoplasts.

15. Add 50 µL of Concanavalin A (concentration 1 mg/ml) to a clean glass-bottom dish to help immobilize the protoplasts while imaging them.

16. Let the Concanavalin A dry for 5 minutes.

17. Add a volume of 3µL diluted protoplasts and let protoplasts settle for 5 minutes before imaging.

18. Examine the protoplasts under the microscope for cell integrity.

Protoplasts vitrification by plunge-freezing – Timing 1 hour

Grid freezing is performed according to Schaffer, M., et al. (Schaffer et al., 2015), with some modifications.

19. Cool down the Plunge Freezer (Leica GP2) and set it to 70% humidity, chamber temperature of 23°C, single backside blotting, 6 sec blot time, and no delay before blotting.

**CRITICAL STEP: Blotting parameters, such as blot time, as well as horizontal and vertical blot positions, need to be determined empirically, as they depend on the actual device used for Plunge Freezing.**

20. Likewise, cool down the cryogen holder for liquid ethane, grid box reservoir, and first grid box to be used.

21. Take 200 mesh gold EM grids holey SiO_2_ foil with tweezers and place them with the side of the foil on an objective glass slide.

22. Place the glass slide containing the EM grids over the Glow Discharge Cleaning System and glow discharge the grids for 90 s at 15 mA. Then, turn the EM grids facing the other side and glow discharge again for 90 s at 15 mA.

23. Store the slide containing the glow-discharged EM grids in a glass petri dish and proceed directly to plunge freezing the protoplasts.

24. Take one of the EM grids with tweezers and place it in blotting position.

25. Pipette 3 µL of diluted protoplasts onto the SiO_2_ side of the EM grid inside the Plunge Freezer.

**CRITICAL STEP: In our experience, the total amount of protoplasts present in 3 µL should be around 1500 to 2000. More or less quantity of cells may promote thick ice formation resulting in insufficient vitrification or not enough plunged protoplasts on the grids, respectively.**

**? TROUBLESHOOTING**

26. Blot the grids from the back-side blotting for 6 sec.

27. Immediately plunge the EM grid containing the protoplasts into the liquid ethane reservoir at -183°C.

28. Then transfer it to the previously cooled-down grid box.

29. Repeat steps 19 to 28 with the rest of the EM grids and store the grid box(es) in a liquid nitrogen storage dewar.

PAUSE POINT: The frozen samples can now be stored in liquid nitrogen storage dewars.

**CRITICAL STEP: A total of 8 - 12 EM grids containing protoplasts can be plunge frozen in 1 hour. On average, in our hands around 70% of all the plunge-frozen EM grids are suitable for the subsequent steps. However, this rate may vary according to protoplast concentration, EM grid material, and glow discharging and plunging conditions.**

**? TROUBLESHOOTING**

Grid clipping – Timing approximately 1 h for about 10 EM grids

30. Once the protoplasts are plunged-frozen, EM grid clipping is carried out similar to a previous protocol of with the protocol of Schaffer, M*., et al.* (Schaffer et al., 2015).

31. Place the clipping support base into the loading box and fill it with clean liquid nitrogen.

32. Once the station has cooled down, transfer the cryo-grid box from the storage dewar to the loading box and open it there.

**CRITICAL STEP: All transfer times should be kept as short as possible to avoid sample contamination.**

**CRITICAL STEP: All tools that will come into contact with the EM grids must be pre-cooled in liquid nitrogen in the loading box before usage.**

33. Mark an AutoGrid with a fine point permanent marker prior clipping, 90° left and 90° right from the milling cut-out. The marks will additionally help to align the lamella sample in the cryo-FIB shuttle for cryo-FIB milling and then in the TEM cassette for cryo-ET.

34. Insert the marked AutoGrid with cryo-FIB cutout into one of the transfer pedestal’s mounting positions.

35. Using a screw driver or comparable tool, loosen the top of the cryo-grid box.

36. Transfer the EM grid from the cryo-grid box and insert it in the Autogrid with the cell side and foil side facing down.

37. Using a loaded C-clip tool, clip the EM grid into the Autogrid with the C-clip.

38. Transfer the clipped EM grid back to the cryo-grid box.

39. Repeat steps 30 to 39 with all the EM grids.

**CRITICAL STEP: Check for bent or damaged grids that are frequently unsuitable for cryo-CLEM or FIB milling and thus should be discarded at this stage.**

Correlative Light and Electron Microscopy (CLEM) for cryo-ET – Timing approx. 1 h per grid.

40. After clipping the EM grids containing the plunge-frozen protoplasts, a fluorescence microscope fitted with a cryo-stage was used for identifying intact root protoplasts expressing RAE1-GFP, while discarding debris and broken/dead cells.

41. Cool down the machine according to the manufacturer’s instructions.

42. Load the clipped EM grid into the cooled microscope

43. Start the software platform for image acquisition, select the proper acquisition parameters, and acquire a grid map overview image.

**CRITICAL STEP: The parameters needed to locate the target of interest will depend on the type of fluorophore with which it is labeled.**

45. Once having the grid map overview, choose and select potential target positions in individual squares.

46. Define and acquire focal-stacks of each of the selected grid squares.

47. The obtained fluorescent images containing the identified target of interest are used for targeted cryo-FIB milling.

Lamellae milling – Timing 8-10 hours, depending on the number of lamellae and sample quality. We usually made 8-10 lamellae during a 10-hour session.

A detailed protocol for loading the grid into the dual beam microscope has already been published (Wagner et al., 2020). Milling lamellae from EM grids is performed with an Aquilos microscope as described previously (Schaffer et al., 2015; Hoffmann et al., 2022).

48. In brief, cool down the dual-beam microscope with liquid nitrogen and allow the stage to reach a temperature of ~ 183˚C. The pressure in the microscope’s chamber should be below 4 x 10^-7^ mbar.

49. Transfer EM grids into the microscope by loading them with pre-cooled tweezers into the shuttle. Make sure that the flat side of the Autogrid containing the protoplasts is facing outwards from the shuttle and orient the cut-out of the AutoGrid towards aligned with the ion beam.

50. Once loaded, check the grids with the SEM at a low current.

51. With the gas injection system (GIS), coat the samples with a layer of organometallic platinum for 10 s.

52. Then, sputter coat the samples with platinum for 10 s at voltage 1kV voltage and 10 mA current.

53. Move to the mapping positions and identify appropriate protoplasts for lamellae milling. Fluorescence data from steps 40-47 can be used as guidance to identify the position of intact protoplasts, which can sometimes be hard to distiguish on the EM grid alone.

**CRITICAL STEP: For compatiblity with cryo-ET later, choose lamellae milling areas that are within 5-6 grid squares from the center of the grid and within the center of individual grid squares.**

54. Once the appropriate milling areas are identified, tilt the stage to a milling position of 15°, corresponding to a lamella angle of 8° in repect to the grid. Begin milling the lamellae in a step-wise fashion, by gradually decreasing the ion beam current from 1 nA, 500 pA, 300 pA and 100 pA.

55. Carry out final polishing of the lamellae with 50 pA to obtain a final thickness between ~180-200 nm of biological material.

56. After polishing, add an optional final sputter layer with platinum for 1-2 s at 1 kV and 10 mA.

**PAUSE POINT: The frozen samples can be stored in liquid nitrogen in their respective storage dewar until imaged by cryo-TEM.**

The following steps are added for completeness. Note that the focus of this protocol is the sample preparation. Cryo-ET acquisition and tomogram reconstruction may vary depending on the software, acquisition scheme used and for different scientific questions.

Automated tomogram acquisition – Timing can vary from hours to days, depending on the number of EM grids and lamellae to be observed, typically we use 2-4 grids for a 48 hr microscope session.

When the desired quantity of milled lamellae is obtained from different EM grids containing plunge-frozen protoplasts, tilt series acquisition can be conducted as described previously (Wagner et al., 2020).

57. Briefly, load EM grids containing milled lamellae into the Autoloader cassette so that the mark on the rim of the EM grid is centered in a 90˚ top-down view, keeping the grids in this position.

58. Load the Autoloader containing the EM grids into the TEM microscope under cryogenic conditions. The marks on the Autogrid help to manually align the grid during loading into the cassette. The cutout should face sideways when loading the cassette.

**CRITICAL STEP: Check the Autogrid orientation to ensure that milling direction of the lamellae are rotated perpendicular to microscope tilt axis. This will ensure access to the areas of interest at high tilt and optimal sample movement when tilting.**

59. Find the lamellae for each EM grid at low magnification and take montaged overview images of the lamellae.

60. Determine the pre-tilt from the lamella orientation and using SerialEM software begin the tilt series acquisition under low-dose conditions.

Tomogram reconstruction – Timing can vary depending on the quantity of data acquired.

Tomogram reconstruction can be performed with adequately established software. We used patch-tracking from IMOD as previously described (Hoffmann et al., 2022).

61. Briefly, pre-process the tilt series by performing dose-filtering using MATLAB (Wan et al., 2017).

62. Remove poor-quality tilt images (if any) from the tilt series.

63. Using eTomo (IMOD, versions 4.11.5), align the dose-filtered tilt series with the patch-tracking alignment method (Mastronarde and Held, 2017).

64. For tomogram generation for visualization purposes, reconstruct back-projected tomograms with SIRT-like filtering.

## Anticipated results

**Figure 5:**
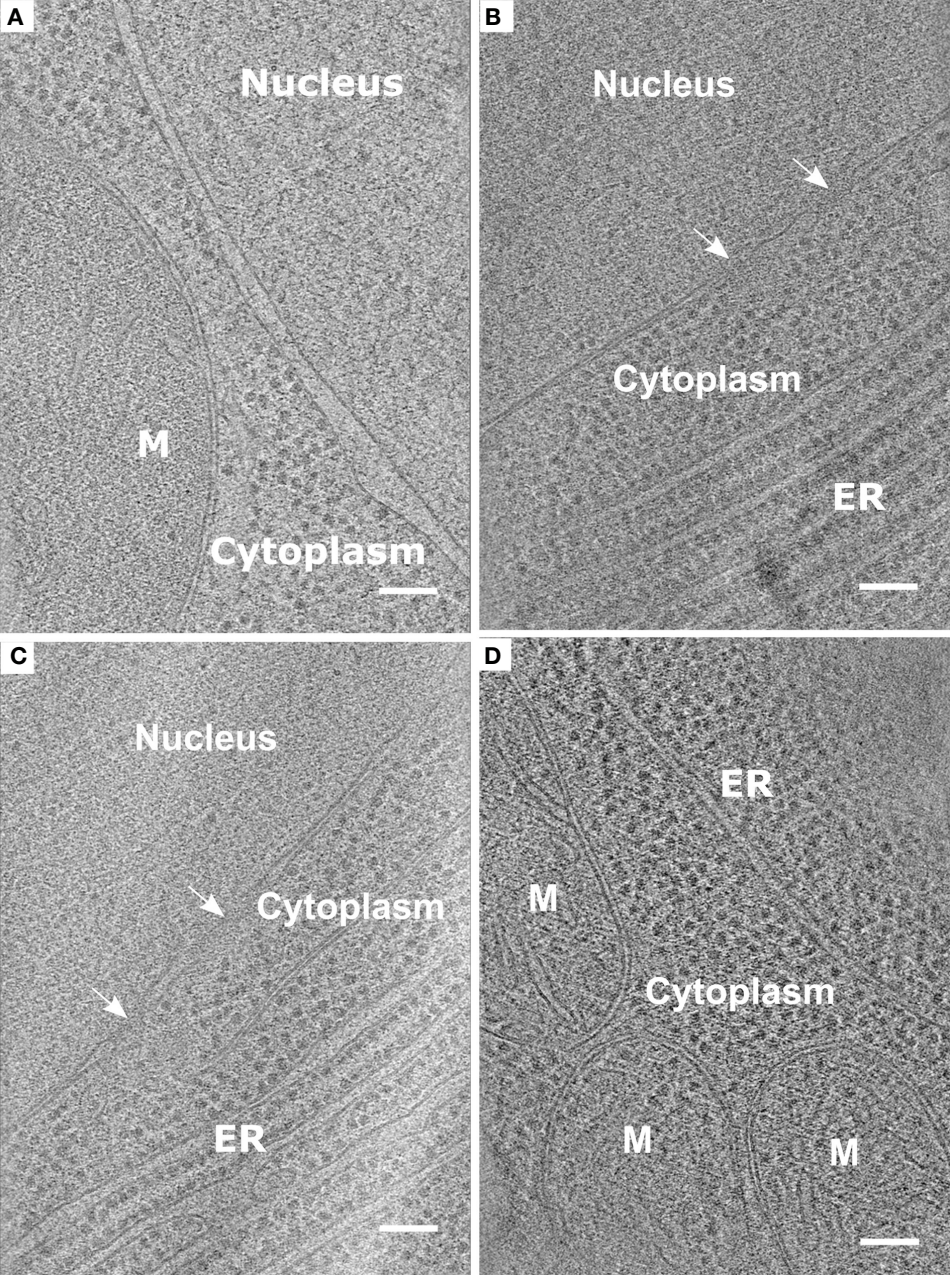
Representative data obtained from the protocol described using root protoplasts from *Arabidopsis thaliana.* **(A-D)**. Slices through tomograms taken from vitrified and milled root protoplasts (lamella thickness: ~ 200 nm or less). Slices through the tomograms show mitochondria (M), Endoplasmic reticulum (ER), and Nuclear Pore Complexes (white arrows). Scale bars: 100 nm **(A–D)**.

Performing the protocol described here will allow the user to prepare and obtain lamellae appropriate for cryo-ET acquisition (results shown in **Figure 5**). Therefore use of protoplasts represents a valuable tool for studying organellar and macromolecular structures in plant cells.

## Troubleshooting

Troubleshooting advice can be found in **Table 1**.

**Table 1:** Troubleshooting table.

Step	Problem	Possible reason	Solution
13	Poor/bad quality protoplast yield	Poor plant growth conditions	Check that the pH of the MS media is between 5.6-5.8. Check and optimize growth conditions, such as light intensity, temperature, and photoperiod.
		Ineffective enzymatic digestion	Optimize digestion time for each transgenic line, accession, and plant tissue.
25	The protoplasts do not adhere properly	Insufficient glow discharging	Verify and adapt glow discharge conditions.
29	Non-vitrified ice in the samples	Many cells were not properly plunge frozen, producing a thick sample	Adjust the number of protoplasts when plunge freezing.
		Poor blotting conditions	Adjust the blotting parameters adequately, depending on the type of filter paper to be used, as well as the blotting force and time.

**TIMING**

Steps 1-2: 2 weeks

Steps 3-18: approximately 2 – 3 h

Steps 19-29: approximately 1 h

Steps 30-39-: approximately 1 h

Steps 40-47: approximately 1 h per grid

Steps 48-56: approximately 8 – 10 h

Steps 57-60: variable timing

Steps 61-64: variable timing

## Data availability statement

The original contributions presented in the study are included in the article/**Supplementary Material**. Further inquiries can be directed to the corresponding author.

## Author contributions

IS: Conceptualization, Formal Analysis, Investigation, Methodology, Writing – original draft, Writing – review & editing. PH: Conceptualization, Data curation, Formal Analysis, Investigation, Methodology, Writing – review & editing, Funding acquisition. TB: Conceptualization, Investigation, Writing – original draft. MB: Conceptualization, Project administration, Resources, Supervision, Writing – review & editing. HG: Conceptualization, Funding acquisition, Investigation, Project administration, Resources, Supervision, Writing – review & editing.

## Supplementary material

Supplementary Video 1 Tomogram showing data obtained from *Arabidopsis protoplasts*. Tomogram taken from vitrified and milled root protoplasts (lamella thickness: ~ 200 nm or less). The tomogram shows mitochondria through the slices. Scale bar is 200nm.
